# Making districts functional for universal health coverage attainment: lessons from Ghana

**DOI:** 10.3389/fpubh.2023.1159362

**Published:** 2023-05-09

**Authors:** Humphrey Cyprian Karamagi, Sokona Sy, Hillary Kipruto, Bertha Kembabazi, Solyana Ngusbrhan Kidane, Thandekile Ntombikayise Moyo, Regina Titi-Ofei, Dominic Atweam, Cornelius Debpuur, Anthony Ofosu, Francis Chisaka Kasolo

**Affiliations:** ^1^World Health Organization—Regional Office for Africa, Brazzaville, Democratic Republic of Congo; ^2^World Health Organization (WHO Zimbabwe), Harare, Zimbabwe; ^3^The Global Fund to Fight AIDS, TB and Malaria, Switzerland, Geneva; ^4^World Health Organization, Accra, Ghana; ^5^Ghana Health Service, Accra, Ghana

**Keywords:** functionality, management, performance, health systems, oversight, service provision assessment, assessment

## Abstract

Ensuring the sub national level in the health system can function effectively is central to attainment of health results in countries. However, the current health agenda has not prioritized how districts can deploy their existing resources effectively, to maximize the efficiency equity and effectiveness in their use. Ghana initiated a self-assessment process to understand the functionality of districts to deliver on health results. The assessment was conducted by health managers in 33 districts during August–October 2022 using tools pre-developed by the World Health Organization. Functionality was explored around service provision, oversight, and management capacities, each with defined dimensions and attributes. The objective of the study was to highlight specific functionality improvements needed by districts in terms of investments and access to service delivery in achieving Universal Health Care. The results showed a lack of correlation between functionality and performance as is currently defined in Ghana; a higher functionality of oversight capacity compared to service provision or management capacities; and specifically low functionality for dimensions relating to capacity to make available quality services, responsiveness to beneficiaries and the systems and three structures for health management. The findings highlight the need to shift from quantitative outcome indicator-based performance approaches to measures of total health and wellbeing of beneficiaries. Specific functionality improvements are needed to improve the engagement and answerability to the beneficiaries, investments in access to services, and in building management architecture.

## Introduction

The 2016 Agenda for Sustainable Development adopted the attainment of good health and wellbeing for all at all ages—its third Sustainable < Development Goal (SDG-3)—as the overarching goal of health actions (1). Given the integrated nature of the SDGs, multiple targets across other SDGs have been identified as contributing to SDG 3. In addition to its 9 core targets and 3 enablers, contributing targets have been identified in other SDGs ranging from 7 to 27 (2–5). The global health community has brought these together under different themes, with the world health organization (WHO) adopting the triple billions approach that structures these into 3 themes relating to attainment of universal health coverage (UHC), health security (HSE) and addressing the determinants of health (DOH) (6). UHC—the umbrella target of the SDG-3 core targets—aims to ensure all persons can access the health and related essential services they need for their health and wellbeing without the risk of financial catastrophe (7, 8). HSE on the other hand brings together all the targets aimed at minimizing negative effects of health shocks while DOH consolidates all the social, economic, environmental, and political targets in other sectors but influencing health and wellbeing.

The global health community has outlined means to translate the SDG-3 goal and targets to implementable actions. A revitalized Primary Health Care approach has been defined as the most effective way to invest in health, for the attainment of UHC, HSE and DOH outcomes (9). This is placing increased emphasis on empowerment of individuals/communities, alignment of stakeholders and integration of services as drivers of how investments need to be made in health (10). Within the WHO Africa Region, a comprehensive menu of actions around which investments need to be made has been elaborated and is guiding country actions (11).

Ghana has been an active contributor to the evolution of this agenda, with the Head of State a co-chair of the UN Secretary Generals (UNSG) eminent group of SDG advocates. The country shared progress towards SDG targets at the 2019 Voluntary National Review process (12), where the slow and uneven progress towards health targets was highlighted. Some targets, such as maternal mortality rate and malaria prevalence were noted to be on the increase. The country defined a UHC roadmap in 2020 (13) that highlighted a number of challenges with moving to UHC. Health service outcomes are only marginally improving, with several gaps seen such as high institutional deaths, inappropriate pediatric HIV response, persisting micro-and under-nutrition deficiencies, increasing noncommunicable diseases all being noted. With the health investments, there is still uneven access to services with low service availability and quality, insufficient staff mix especially in primary care facilities, lack of basic infrastructure in over half of primary care facilities, unstable external financing due to lower middle income country (LMIC) progression, high exposure to out of pocket expenditure and only 20% of insurance expenditures incurred at primary level facilities.

The country has a strong history of health reforms, to align sector focus with expectations. The administration has been decentralized since 1993 with de-concentration of national functions to a current 16 regions, and devolution of responsibilities to a current 261 Metropolitan, Municipal and District Authorities (14). Health services management has been delegated to an autonomous agency of the Ministry of Health, the Ghana Health Service since 1996 (15). Since 2001, there have been formal health sector performance reviews to document progress being made against pre-determined indicators at national and regional levels (16). A National Social Health Insurance Scheme was established in 2003 (17), which was replaced with the National Health Insurance Act in 2012 to better address financial risk protection challenges by eliminating the ‘cash and carry’ system of paying for healthcare (18). To align with the current health reforms particularly in relation to a revitalized PHC approach to attain UHC and other health goals, the country in 2020 developed a UHC roadmap to channel efforts towards attaining its desired results by 2030 (19). Part of this involves initiation of institutional reforms particularly at the district level, to enhance efficiency and effectiveness in provision of essential services.

While the country is reliant on the regions and districts in coordination and management of the delivery of desired health results, there are no tools or processes to inform how well these are functioning (20). Functionality here is focusing on how well the region/district is deploying its existing resources, to facilitate attainment of its desired results. The existing processes focus on assessing performance—which focuses on comparing a unit of performance against pre-determined norms such as a coverage target. This information, while useful for a high-level decision maker, is of limited utility to a mid—frontline manager who is concerned with whether they are appropriately deploying their available resources. Functionality of the sub national unit in this context is critical as it represents the first point of action that will lead to attainment of the desired health results—it has been documented to strongly correlate with attainment of desired health results (21). The objective of the study was for Ghana to embarked on a process to of determining how well the districts and regions are functioning. The aim was to work with health management teams at the regional and district level to identify areas where they need to place emphasis, to improve the overall functionality of the districts/regions. This it is presumed will facilitate accelerated attainment of UHC, HSE and other health results in Ghana. We present the methods and outputs from this process.

## Methodology

### Purpose

The overall purpose was to systematically review the functionality of the district system in Ghana. The output would highlight specific areas for a participating district to focus on, while concurrently identify emerging areas for prioritization at the regional and national level.

### Site

The assessment was conducted in a sample of districts, focusing on those in six newly established regions of the country by a country team (22). A focus on the districts in newly established regions was to provide these new districts with guidance on where to prioritize their focus to accelerate their contribution to the country’s health results.

### Process for review

The assessment using the WHO tool for reviewing the functionality was conducted during August–December 2022. A 10-member national planning and implementation team was constituted on 8 August 2022 to review the tool and adapt the tool to reflect the country context The members included health research scientists, district directors of health services, regional directors of health services, regional and district health information officers, health planners, public health specialists and public health nurses. Review tools were pretested during 23rd August–7th September in the Ga East and Ningo Prampram districts. Following incorporation of lessons from the pre-test, the preparation and review in the review districts was conducted concurrently, between 24th October–end December 2022.

### Sample selection

A target of five districts in each of the 6 new regions were identified to participate in the assessment. Based on their high and low performance on their attainment of a basket of health outcomes in the year 2021 (outcomes were coverages for ANC 4 +; Penta3; % HIV + ves on treatment; and TB treatment success rate) 18 districts in total were included. Data on the indicators was extracted from DHIMS as of August 12, 2022. Based on the regional average for each indicator, a district is given a score of 1 if it performed above the average, 0 if equal to the average, and-1 if less than the average. The overall performance score for each district was obtained by summing the scores for the four indicators. The overall score ranged from – 4 to + 4.

### Review team

In each district, a multidisciplinary team of at least 6 members was formed (see Supplementary Appendix 1 for members and their designation). In each region, a 4-day orientation meeting was organized for all the selected district members, where participants were oriented on the process and tools. Each district team worked as a unit to conduct their own district review, under observation of the national team members present to ensure queries were clarified, and each district team member was actively engaging in the process. Self-assessment was the best methodology in this case because the process aim for both actions to be undertaken by the managers and providing information for cross district comparison. The review tools were designed for this purpose in mind.

### Review tool

The tool used in the review were developed by the WHO Regional Office for Africa.^1^ It is an attribute-based tool that allows self-review of functionality across oversight, management, and service provision capacities at a specific sub national unit. Several dimensions construct each of the above capacities, against which the attributes are described (see Supplementary Appendix 2 for the working definitions of each dimension and their full set of attributes). Oversight capacity reviews functionality of the decision-making process across 6 dimensions drawn from governance literature (23–25): organizational structure, policy and strategic guidance, technical and social accountability, legal and regulatory frameworks, stakeholders’ engagement, and integrity and public confidence. Management capacity reviews functionality of the implementation team across 7 dimensions drawn from management literature (26–28): structure, strategy, systems, managerial style, skills, staff capacities and values. Finally, the service provision capacity reviews functionality of the system investments needed to provide services across 4 dimensions drawn from service delivery functionality literature (21): overcoming access barriers, quality of care, demand for essential services and resilience of the system.

For each dimension above, a set of attributes are defined that describe what the sub national unit is expected to do. The review by the sub national unit is to determine, on a Likert scale, where it lies regarding the description of the attribute. The sub national unit scores the attribute as 1 (fully disagree), 2 (somewhat disagree), 3 (somewhat agree) or 4 (fully agree) depending on its situation as agreed by the review team. A ‘not applicable’ option also exists in case the sub national unit does not consider the attribute applicable.

### Analysis of review information

The review is meant to be of primary use for the sub national unit participating. As such, the sub national unit receives a consolidated list of attributes organized by the score immediately the assessment is completed. This allows the SNU to immediately know and plan action, to enhance its performance as the attributes are described in a manner that necessitates action. For the attributes scoring poorly (score of 1), it suggest actions to implement the attribute, while it needs a plan to sustain attributes where it scores well (score 4). For those where a SNUscores ‘NA’, the SNU needs to explore why it is not applicable and consider their implementation.

To discern a regional and national picture about functionality in the newly created regions, we conducted several statistical tests on the data from all the districts. Prior to analysis, we first calculated an arithmetic mean for the values of the attributes constructing each dimension by district. The values were then converted from a 0–4 to a 0–100 scale by applying a multiplication factor of 25. We then tested the district data for compliance to assumptions for an analysis of variance using Bartlett’s Chi-square test, with the results showing significant homogeneity of variances.

First, we explored variations in findings between the high (*n* = 7) and low (*n* = 11) performing districts. We looked at the mean, standard deviation, and confidence intervals, together with analysis of the between and within group variations using analysis of variance (ANOVA) to determine if there were differences in the two groups. *F*-statistic and associated value of p were used to determine the level of significance.

Second, we explored the variations in findings across the 3 capacities of functionality—service provision, oversight, and management capacities. Each, conducted one way analysis of variance (1-way ANOVA) focusing on the mean, standard deviation, 95% confidence interval for the reporting districts (*n* = 33). We further explored the source of variation both between and within the 3 capacities of functionality to determine the statistical significance, if any, of reported variations. To explore the multiple possible comparisons amongst the 3 different capacities, we applied the Scheffé test (29) to explore the significance of variation between (1) Management and oversight capacity, (2) management and service provision capacity, and (3) oversight and service provision capacity. A statistically significant value (*p* < 0.05) would be interpreted to mean significance in the difference between these capacities.

Third, we analyzed the variations in findings across the dimensions constructing each of the 3 capacities of functionality. For each capacity, we conduct one way analysis of variance focusing on the mean, standard deviation, 95% confidence interval for the 33 reporting districts. We further explore the source of variation both between and within the dimensions for each capacity to determine the statistical significance if any of the variations reported. We apply the Scheffé test to explore the significance of variations across the different combinations of dimensions making up each capacity—with a statistical significance value suggesting significance between the combinations.

Finally, the mean attribute scores were sorted and ranked according to the scores from the districts, to classify them based on numbers of districts scoring each value. This is to identify the most reported attributes as being (1) not applicable, (2) most functional, and (3) least functional.

The script for the analysis has been uploaded on the github and can be accessed publicly at: https://github.com/DAK-Projects/SNU_Ghana.

### Presentation of results

Presented first were the overall findings and followed by presentation of each of the 3 capacities for the districts. After this, we presented results from the statistical analysis of the results at three levels: for different levels of district performance; for the different capacities and for the dimensions constructing each capacity. We lastly presented the five attributes reporting the most and least functionality across the reporting districts.

## Results

### Overall functionality of districts in Ghana

The overall reported level of functionality by district and for each functionality capacity are displayed in Table 1. We present for each district its performance classification prior to the study, together with the results for its functionality, overall and by each of the capacities that construct it. The functionality by dimension is shared in Supplementary Appendix 3. The reporting districts have a mean was 80.68, with the functionality of the oversight capacity dimensions highest and that of the service provision capacity lowest.

**Table 1:** Overall functionality and by capacity for the 33 reporting districts in Ghana, 2022.

District name	Performance classification	Overall functionality	Service provision capacity	Management capacity	Oversight capacity
Juan	High	73.35	70.57	54.33	95.14
Nkwanta South	High	90.20	83.85	93.00	93.75
Nkwanta North	High	57.39	53.13	42.67	76.39
Tano North	High	91.21	87.24	93.33	93.06
Asutifi South	High	87.58	85.16	88.00	89.58
Nkoranza South	High	77.03	69.27	74.33	87.50
Bunkpurugu Nakpanduri	High	62.56	58.07	64.33	65.28
Krachi West	Low	87.38	86.72	81.67	93.75
Krachi Nchumuru	Low	76.69	78.91	55.33	95.83
Asunafo South	Low	91.03	88.28	89.67	95.14
Tano South	Low	79.25	75.78	79.33	82.64
Asutifi North	Low	83.82	80.47	79.33	91.67
Kintampo North Municipal	Low	89.89	84.90	90.33	94.44
Techiman Municipal	Low	73.10	73.96	70.33	75.00
Pru	Low	84.39	79.95	84.33	88.89
Yunyoo-Nasuan	Low	67.72	61.98	53.67	87.50
Chereponi	Low	91.49	87.24	90.00	97.22
East Mamprusi	Low	77.26	75.26	76.67	79.86
Ga East	NA	70.50	65.36	67.67	78.47
Ningo Prampram	NA	76.29	72.66	75.67	80.56
Bia East	NA	77.13	79.17	63.33	88.89
Bodi	NA	69.18	64.06	48.33	95.14
Mamprugu Moagduri	NA	72.63	58.07	73.00	86.81
Aowin	NA	79.47	72.92	78.00	87.50
West Mamprusi Municipal	NA	95.36	95.31	96.33	94.44
Bole	NA	76.69	71.09	76.33	82.64
North East Gonja	NA	80.80	63.02	86.33	93.06
East Gonja	NA	96.72	95.57	96.67	97.92
Central Gonja	NA	93.32	92.71	89.33	97.92
North Gonja	NA	72.36	66.41	50.67	100.00
Sawla-Tuna-Kalba	NA	93.46	86.20	97.67	96.53
West Gonja	NA	87.14	81.51	82.00	97.92
Sefwi Akontombra	NA	79.88	76.30	80.00	83.33
Total for reporting districts		80.68	76.40	76.42	89.20

### Functionality across different levels of performance

The mean for the high performing districts is lower than that for the low performing districts (74.93 vs. 80.37). The statistical analysis for these districts is shown in Table 2.

**Table 2:** Overall mean functionality score for districts in newly created regions of Ghana in 2022.

Groups	Sample	Sum	Variance	Std Dev	**Mean**
All districts	*33*			*13.20*	80.68
High performing districts	7	524.52	209.81	14.49	74.93
Low performing districts	11	884.06	70.76	8.41	80.37
Source of Variation	*d.f.*	*SS*	*MS*	*F*	value of *p*
Between groups	1	126.50	126.50	1.029	0.325
Within groups	16	1,966.42	122.90		
Total	17	2,092.92			

**Figure 1:**
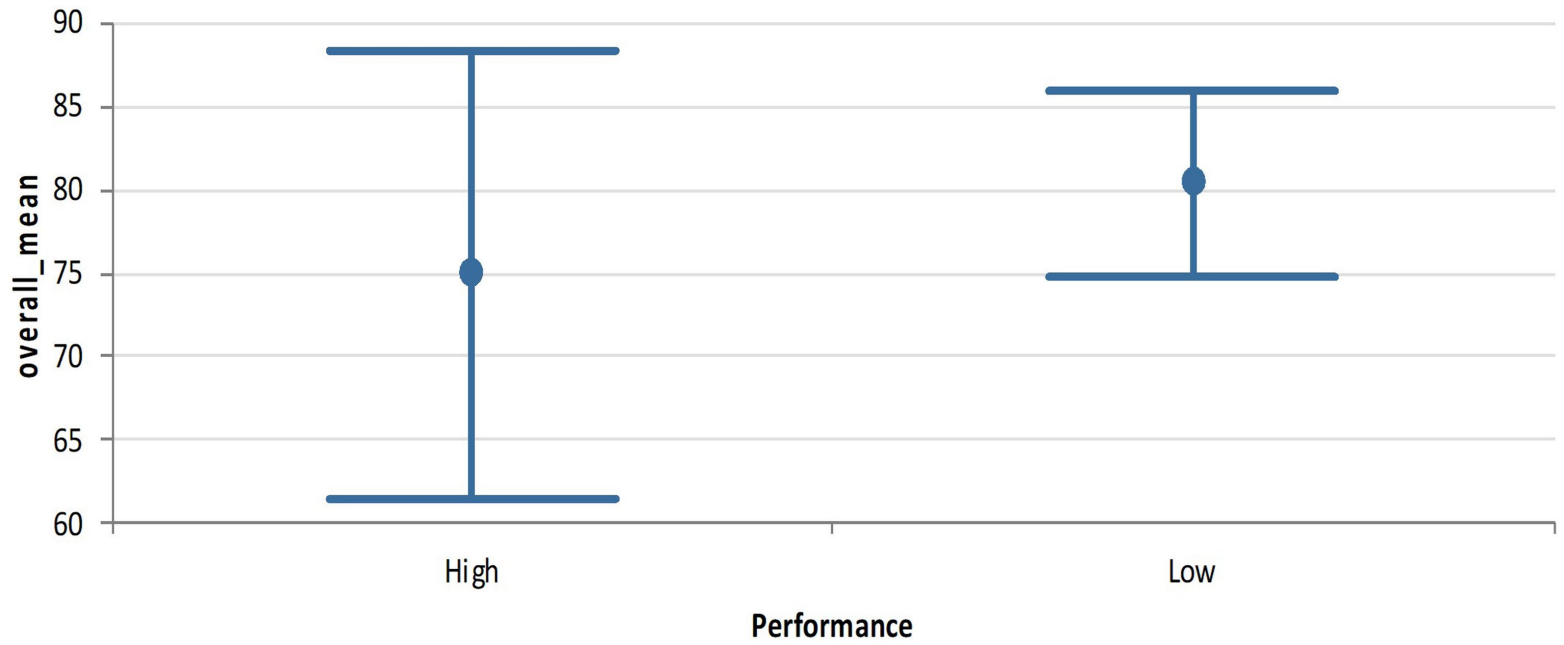
Means plot at 95% confidence interval for high and low performing districts in Ghana.

The variation in functionality by performance is not statistically significant, with a low value of p for this variance (0.325). We see in Figure 1 below that the confidence limits significantly overlap, with the low performing districts confidence limits within the range of that for the high performing districts.

### Functionality of the capacities contributing to the overall picture

Looking at the capacities contributing to this overall functionality summarized in Table 3, oversight capacity is reported to be highest (89.2), while the service provision is lowest (76.42). These variations across the 3 capacities are statistically significant (*p* = 0.000115).

**Table 3:** Mean score for constituent capacities of district functionality in newly created regions of Ghana in 2022.

Capacities	Sample size	Std Dev	Mean	95% confidence interval	
Management capacity	33	15.22	76.42	71.03	81.82
Oversight capacity	33	8.09	89.2	86.34	92.07
Service provision capacity	33	11.13	76.4	72.45	80.34
Source of Variation	*d.f.*	*SS*	*MS*	*F*	*Value of p*
Between capacities	2	3,601.133	1,800.567	12.829	0.000
Within capacities	96	13,473.826	140.352		
Total	98	17,074.959			
*Scheffe group vs. Group (Contrast)*	*Difference*	*95% confidence interval*	*Test statistic*	*Value of p*	
Management vs. oversight capacities	(12.780)	(20.032)	(5.529)	4.382	0.000
Management vs. service provision capacities	0.027	(7.224)	7.279	0.009	1.000
Oversight vs. service provision capacities	12.808	5.556	20.060	4.391	0.000

**Figure 2:**
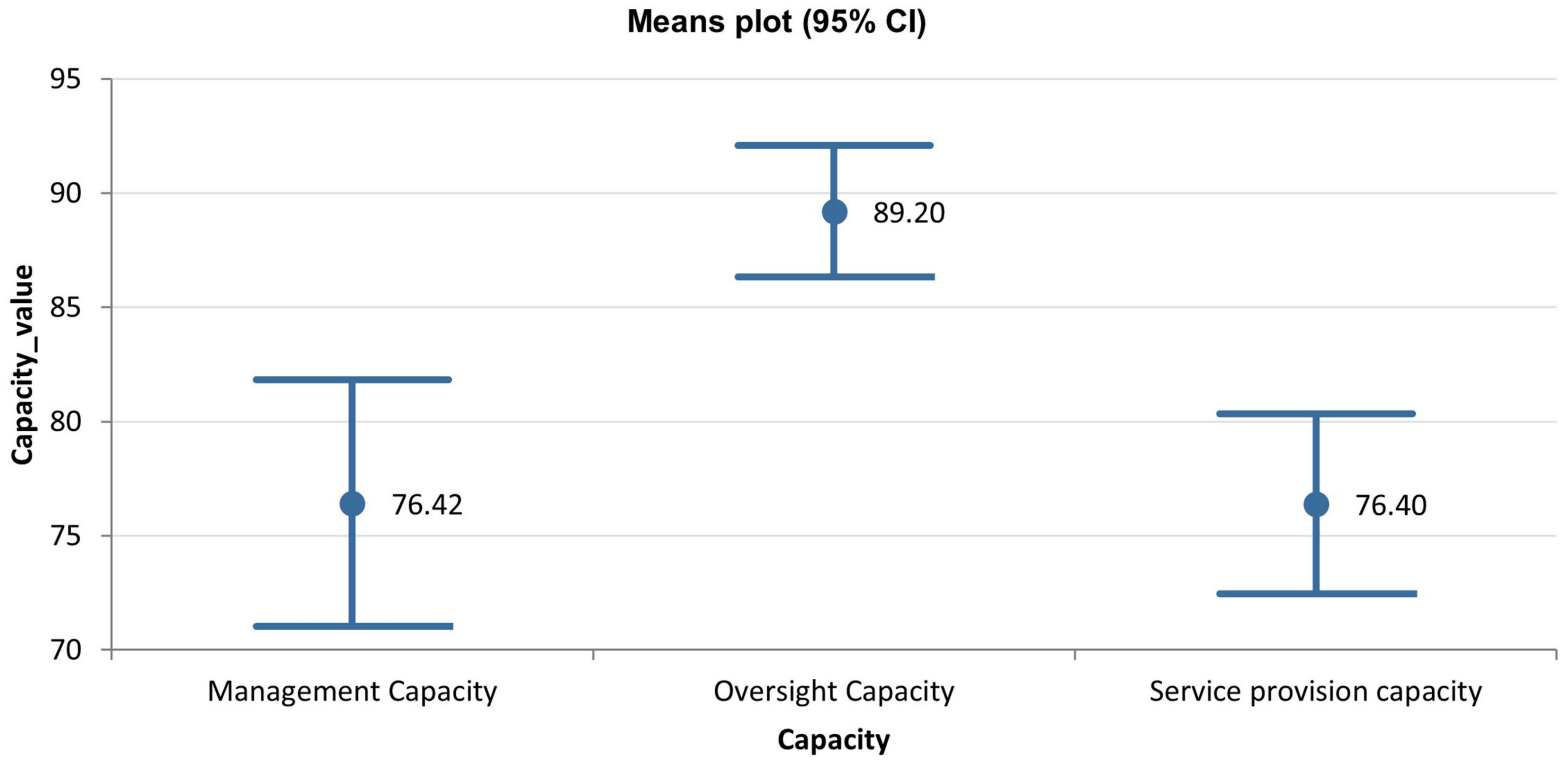
Means plot at 95% confidence interval for the 3 functionality capacities in Ghana.

However, the variation between the management (76.42) and service provision (76.40) capacities is not statistically significant; there is considerable overlap between the confidence limits of these two capacities, as shown in Figure 2 below.

### Functionality of the dimensions contributing to each capacity

Within the specific capacities, functionality was driven by specific dimensions, as illustrated in Table 3. The variations in the dimensions for all capacities show statistical significance. For service provision, the functionality for different dimensions is, in descending order, (1) demand for services, (2) system resilience, (3) quality of care, and (4) access to essential services. Looking at oversight capacity, the dimension functionality in descending order is (1) technical accountability, (2) stewardship, (3) policy/strategic guidance, (4) authority and mandate, (5) legal/regulatory framework, (6) stakeholder engagement, (7) integrity and public confidence, and (8) social accountability. Finally with the management capacities, the dimensions functionality in descending order is (1) strategy, (2) leadership style, (3) shared values, (4) appropriate staff, (5) required skills, (6) required managerial structure and (7) needed systems (Table 4).

**Table 4:** Mean scores for constituent dimensions for functionality capacities in newly created regions of Ghana in 2022.

Capacity	Feature of analysis	Outputs				
Service provision	*Dimension*	*Sample size*	*Std Dev*	*Mean*	*95% confidence interval*	
	access	33	17.88	68.89	62.55	75.23
	demand	33	11.75	89.77	85.60	93.94
	quality	33	13.69	76.85	71.99	81.70
	resilience	33	12.57	77.46	73.00	81.92
	*Source of variation*	*d.f.*	*SS*	*MS*	*F*	Value of *p*
	Between groups	3	7,361.02	2,453.67	12.22	0.0000
	Within groups	128	25,701.76	200.79		
Oversight capacity	*Dimension*	*Sample size*	*Std Dev*	*Mean*	*95% confidence interval*	
	Authority	33	8.90	91.41	88.26	94.57
	Engagement	33	14.55	86.74	81.58	91.90
	Integrity	33	15.35	82.39	76.95	87.83
	Legal	33	13.17	90.34	85.67	95.01
	Policy	33	11.91	91.82	87.59	96.04
	Social	33	18.45	79.36	72.81	85.90
	Stewardship	33	9.39	94.09	90.76	97.42
	Tech	33	7.12	94.51	91.98	97.03
	*Source of variation*	*d.f.*	*SS*	*MS*	*F*	Value of *p*
	Between groups	7	7,043.38	1,006.20	6.09	0.0000
	Within groups	256	42,282.62	165.17		
Management capacity	*Dimensions*	*Sample size*	*Std Dev*	*Mean*	*95% confidence interval*	
	skills	33	20.81	74.65	67.27	82.03
	staff	33	18.02	78.79	72.40	85.18
	strategy	33	10.56	90.78	87.04	94.53
	structure	33	16.97	72.58	66.56	78.59
	style	33	13.26	81.25	76.55	85.95
	systems	33	19.26	68.09	61.26	74.92
	values	33	19.81	81.06	74.04	88.08
	*Source of variation*	*d.f.*	*SS*	*MS*	*F*	Value of *p*
	Between groups	6	10,647.62	1,774.60	5.93	0.0000
	Within groups	224	67,080.29	299.47		

The confidence limits overlap for many of the dimensions, despite the significant differences. From the Scheffé test (see Supplementary Appendix 2), we see significant differences between dimensions that are not sharing the same confidence limits. For the service provision capacity, these are demand for services against all the other dimensions. With the oversight capacity, we see significance in the value of ps for (1) integrity and public confidence versus stewardship and technical accountability, and (2) social accountability versus authority/mandate, policy/strategic guidance, stewardship, and technical accountability. With the management capacity, significance is seen in value of ps for strategy versus skills, structure, and systems.

### Attributes of specific mention

Finally, looking at the specific attributes within each dimension, we identified the 5 attributes that were mostly mentioned as available or not available in the districts. For the attributes most captured as not being done were:

– Presence of health equipment (blood pressure apparatus, stethoscope, adult and infant scale, thermometer) in 80% of primary care units,
– Presence of health facilities as per norms,
– Outreach and mobile services conducted into the hard-to-reach regions from hospitals,
– Monitoring, management and reporting of radiation emergencies, and
– Presence of clinical support staff in hospitals.

On the other hand, the five attributes most reported as being there but inadequate were:

– Presence of primary care staff with technical skills to treat, rehabilitate and provide palliative care for users with cancer,
– Use of online consultations by hospitals to improve capacity and access for beneficiaries,
– Conduct of emergency simulation exercises to assess capacity to respond to potential shock events,
– Monitoring, management and reporting of radiation emergencies, and
– Presence of hospital staff with technical skills to treat, rehabilitate and provide palliative care for users with cancer.

Finally, the five attributes most reported as being there and adequate were:

– Primary care facilities have the expected service provision areas.
– Mechanisms for health leadership to answer/report on their progress, e.g., through performance monitoring.
– Presence of community level service delivery modalities (home visits, community outreaches, community events, etc.).
– Outreach and mobile services conducted into the hard-to-reach regions from primary care facilities.
– Availability of programs for promoting health and wellbeing for under 5 s.

## Discussion

The results highlight the importance of having a clear distinction between functionality—how well resources are being deployed for attainment of desired results, and performance—what results are being attained. It is common to presume they are synonyms and correlated in practice, but this is not so. It is common to perceive performance in line with coverages achieved for selected indicators—in this case antenatal care, immunization, HIV and TB care being the selected ones. Significant investments have been focused on refining and improving health system performance frameworks that focus on identifying indicators that better reflect dimensions relating to effectiveness, equity, quality of care, and/or efficiency (20, 30–31). The use of performance measures based on selected outcome indicators may not be adequate for informing progress at the district level for two reasons: (1) it under-estimates the range of outcomes actually achieved from a functional system, and (2) it largely focuses on the quantitative and easy to get indicators, skewing the perception of performance. The current health results such as UHC define desired outcomes not on how many program outputs are delivered, but rather by their impact on the beneficiary—ensuring everyone, everywhere is getting services they need for their health and wellbeing while avoiding financial catastrophe. A functionality review such as the one reported here is a better pointer to whether a system will attain UHC and other person-focused health results that are currently desired, as they present a picture of the status of the range of needed actions for health managers to focus on to attain the results. The absence of correlation between functionality and the way performance is currently measured is therefore expected. An interpretation of performance based on consolidating vertical programmes outcomes is at best an incomplete view of performance arising from investment made in the health sector. The current health sector performance approaches that are based on selected programmes outcomes—usually child and maternal health outcomes—grossly under-represent the wide range of outcomes that arise from investment in health. When we look at the capacities constructing functionality, we see the managers view a higher level of oversight capacity compared to service provision and management. Oversight is concerned with the capacity of the district to engage with, show direction and respond to the expectations of its beneficiaries and stakeholders. This higher capacity may reflect the emphasis that has been placed on the oversight functions vis-à-vis the service provision and management. It is common for support programs—whether public or donor—to have significant budgets for different oversight functions compared to the other capacities. If health results are to be attained, it is important for more focus and investment to be placed on management and service provision capacity at the district level.

A further analysis of the oversight dimensions show there is a higher perception of functionality for those focusing on ensuring clear direction such as technical accountability, stewardship, policy/strategic guidance with less emphasis on those dimensions relating to beneficiaries such as ensuring stakeholder engagement, integrity and public confidence plus social accountability. Managers appear to ensure functionality in being seen to be ‘doing the right thing’ in the eyes of whoever is watching, compared to ‘doing things right’ in the eyes of the beneficiaries. As such even though oversight capacity appears to be most functional, there is still immense scope for improvement particularly in relation to how the beneficiaries engage with and contribute to the direction being taken.

When we look at service provision capacity, it is concerned with the capacity to ensure the population in the district is, during both routine and emergency situations, accessing quality essential services they are demanding for their health and wellbeing. The functionality of this capacity is intuitively the most central as it represents the ability to deliver the needed services to the population. We however see the dimension of demand as being the most functional, while access to essential services is least functional. This presents an interesting dichotomy in engagement with beneficiaries—while at the service provision (facility/community) level services are being tailored to the demands from the beneficiaries, we noted at the oversight level that there is limited functionality of initiatives to ‘do things right’ in the eyes of the beneficiaries. The means of engaging with beneficiaries at the governance level are not linked to the service provision level. This may contribute in practice to the limited interest of beneficiaries in engaging with oversight and governance institutional arrangements, even where these exist—with many institutional arrangements resorting to coercive approaches to be able to function (32). On the other hand, the low functionality of access to essential services mirrors the current evidence, where physical, financial and sociocultural gaps are documented as being the rate limiting step in accelerating movement towards desired health results (21, 33). Interestingly, the capacity attributes relating to system resilience are viewed higher than access or quality of care. This is primarily due to the higher assessed levels of inherent system resilience within the Ghana districts—that aspect of resilience to do with the inherent nature of the system to anticipate, absorb, adapt to and transform when faced with unplanned shock events (34–36). Investments in targeted resilience—focused on known shock events—are still quite low in the districts. Finally with the service provision capacities, the quality-of-care attributes are only better than access to essential services. The importance of quality of care cannot be under-estimated, as it addresses crucial areas relating to the process of care provision (37).

Finally, when we explore the results relating to the management capacity, we again see some interesting trends. The dimensions relating to the ‘architecture of management’—such as the needed systems and structures are least functional, relative to those relating to the software subjective management dimensions such as strategy, leadership styles and shared values. Many of the existing management programs focus on these subjective management dimensions particularly on planning, change management methods and leadership skills, with little emphasis on ensuring the needed architecture of management exists and is functional (38, 39).

## Limitations of the analysis

The results and discussion represent findings from self-assessments carried out by health managers in 33 districts. The implications of the study need to be interpreted with this in mind. Self-assessments are prone to assessor bias. However, we feel the impact of this on the results is reduced by the expensive training done prior to the assessment, large number of different officers involved in the assessment in each district and the emphasis during entry of data on the fact that the results were primarily for local use—and not a traditional data collection exercise.

Additionally, the number of districts (32) involved in the sample were few compared to the current 261 Metropolitan, Municipal and District Authorities in Ghana. The districts were purposively selected—as these were newly created districts and were not aimed at being representative for the whole country. Again, the results need to be looked at in this perspective.

## Implications and conclusion

The functionality of districts does provide unique information not only for the district as it self-assesses itself, but also at the management level to know where to channel support if the district is going to meaningfully contribute to the health results the country is aspiring to. From this analysis, we have identified some critical areas of emphasis needed to make the districts better functioning for the delivery of UHC and other related health results.

First, we see a need to rethink the way we are measuring performance particularly at the district level. We need to shift from quantitative outcome indicator-based measures, towards those that measure the total health and wellbeing of beneficiaries as this is the best way to capture the wide range of outcomes achieved from functional systems.

Second, we need to focus on enhancing specific aspects of oversight, management, and service provision capacities at the district level. With oversight capacity, focus is needed on improving engagement and answerability to the beneficiaries. With service provision capacity, access to services remains a major bottleneck to functionality with more investments needed in ensuring the staff, medicines/supplies and infrastructure exists for beneficiaries to reach services they need. The management on the other hand need to invest in building the management architecture and not only focus on the software.

At present, the country is expanding the use of the functionality tools to all districts as they have found it a useful tool for determining attributes on which to focus actions during their planning process. The same process is being conducted in other countries, which should facilitate alignments of the way districts are organized and managed with their current expected results particularly in relation to attainment of UHC, health security and improvements in the determinants of health.

It is our opinion that further research would help to better understand the patterns being seen in functionality and how to link this to a broader view of performance of health systems using appropriate indicators that cover the breadth of areas impacted by actions in health.

## Data availability statement

The original contributions presented in the study are included in the article/Supplementary material, further inquiries can be directed to the corresponding author.

## Author contributions

HuK, AO, and FK share senior authorship. CD, AO, and FK share last authorship. HuK, SS, and SK led the design of the study. SK, DA, and CD coordinated the fieldwork and district engagements. SS, HiK, and BK led the analysis of the data. DA, CD, AO, and FK led the review and contextualization of the work within Ghana. HiK, AO, and FK led the interpretation of findings and discussion. All authors contributed to the article and approved the submitted version.

## Conflict of interest

The authors declare that the research was conducted in the absence of any commercial or financial relationships that could be construed as a potential conflict of interest.

## Publisher’s note

All claims expressed in this article are solely those of the authors and do not necessarily represent those of their affiliated organizations, or those of the publisher, the editors and the reviewers. Any product that may be evaluated in this article, or claim that may be made by its manufacturer, is not guaranteed or endorsed by the publisher.

## Footnotes

^1^ http://bit.ly/3XHFXb4
