# Supplementary material for: Making districts functional for universal health coverage attainment: lessons from Ghana
Source: Front Public Health. 2023 May 9;11:1159362. doi: 10.3389/fpubh.2023.1159362 (PMC10204803; doi:10.3389/fpubh.2023.1159362)
Supplement: Supplementary file 1 [file Table_1.DOCX]

# Supplementary Appendix 1: District Assessment Teams

| **No** | **District** | | **Name** | **Designation** | **Dept/Unit** |  |
| --- | --- | --- | --- | --- | --- | --- |
| **Ahafo Region** | | | | | | |
| 1 | Asunafo South | | Sampson Acquah | DPO | District Assembly |  |
| 2 |  |  | Isaac Yeboah | HIO | DHD |  |
| 3 |  |  | Asante Offei | PA | Sankore HC |  |
| 4 |  |  | Jeremiah Tankoruk | Ag. PHN | DHD |  |
| 5 |  |  | Kwadwo Ansong | DDHS | DHD |  |
| 6 |  |  | Jasper Domack | PHN | DHD |  |
| 7 | Tano South | | Kofi Adomako | ADPO | District Assembly |  |
| 8 |  |  | Emmanuel Baah | HIO | DHD |  |
| 9 |  |  | Dr. Agyei Darko E. | Med. Supt | Bechem Hospital |  |
| 10 |  |  | Grace Amponsah | PHN | DHD |  |
| 11 |  |  | Hayford Baah | RGN | Derma Health Centre |  |
| 12 |  |  | Samuel Kojo Dadson | HPO/DDHS | DHD |  |
| 13 | Tano North | | Eva Anyea | DDHS | DHD |  |
| 14 |  |  | Abena Konadu Gyimah | PHN | DHD |  |
| 15 |  |  | Musah-Abdul Wahab | DPO | Municipal Assembly |  |
| 16 |  |  | Isaac Owusu | HIO | DHD |  |
| 17 |  |  | Deboarah Adams | PA | Yamfo HC |  |
| 18 |  |  | Dr. David Twumasi | Med. Supt | District Hospital |  |
| 19 | Asutifi North | | Belinda Boateng | HIO | DHD |  |
| 20 |  |  | Danso Seth Andrew | ADPO | District Assembly |  |
| 21 |  |  | Dr. Daniel Okine | Med. Supt | Kenyasi Hospital |  |
| 22 |  |  | Juliana Amankwaah Mensah | PNO (PH) | DHD |  |
| 23 |  |  | Charlotte Adu Gyamfi | DDHS | DHD |  |
| 24 |  |  | Anthony Nwineebo | Pharm | Gyedu HC |  |
| 25 | Asutifi South | | Joshua Siaw | HIO | DHD |  |
| 26 |  |  | Dr. Ivan Muanah | Med. Supt | St. Elizabeth Hospital |  |
| 27 |  |  | Foster Nyarko | DDHS | DHD |  |
| 28 |  |  | Christian Abotsie | PHN | DHD |  |
| 29 |  |  | Mercy Sackey | Midwifery officer | Acherensua HC |  |
| 30 |  |  | Sabina Obeng | Planning officer | District Assembly |  |
| **Bono East Region** | | | | | | |
| 1. | Kintampo North | Dr. Collins Boateng Danquah | | DDHS | MHD |  |
| 2. |  | Nanga Joseph | | HIO | MHD |  |
| 3. |  | Juliana Aboud | | Public Health Nurse | MHD |  |
| 4. |  | Tukuu Eric | | Administrative Manager | MHD |  |
| 5. |  | Adama H. Lukeman | | EPI Coordinator | MHD |  |
| 6. |  | Dr. Ofori William | | Med Sup | KMH |  |
| 7. |  | Seth K. Antwi | | Administrative manager | KMH |  |
| 8. |  | Grace Burema Excellence | | Nurse Manager | KMH |  |
| 9. |  | Bright Gyamfi | | Physician Assist. | Asantekwa CHPS |  |
| 10. | Techiman Municipal | Dr. Kwabena Fosuhene Kusi | | MDHS | MHD |  |
| 11. |  | Dr. Fadle Rahman Quantson | | Med-Supt | Abrafi Women and Children Hospital |  |
| 12. |  | Hyacinth Kuupuolo | | DDNS | MHD |  |
| 13. |  | Cosmas Bakyeayiri | | Senior Field Technician | MHD |  |
| 14. |  | Stephen Baidoo | | Administrative Manager | MHD |  |
| 15. |  | Osei-Wusu Sadick | | Public Health Officer (Hi) | MHD |  |
| 16. | Nkoranza North | Ernest Kyeremeh | | District Disease Control Officer | DHD |  |
| 17. |  | Yussif Reyadudeen | | District Public Health Nurse | DHD |  |
| 18. |  | Sieh Daniel | | District Health Information Officer | DHD |  |
| 19. |  | Awiti Stephen | | District Mental Health Officer | DHD |  |
| 20. |  | Dr. Deborrah Danso | | Ag. Medical Officer | Busunya Polyclinic |  |
| 21. |  | Dr. Bismark Osei Amankwaa | | Pharmacist | Busunya Polyclinic |  |
| 22. | Nkoranza South | Dr. Emmanuel A. Teviu | | Mun Director of Health Services | DHD |  |
| 23. |  | Ms. Sylvina Twenewaa | | Public Health Nurse | DHD |  |
| 24. |  | Sampson Kormla Addo | | Health Information Officer | DHD |  |
| 25. |  | Emmanuel Takyi Adjei | | Administrative Manager | DHD |  |
| 26. |  | Dr. Rosemond Kokuro | | Medical Director | St Theresa Hospital |  |
| 27. |  | Dr. Atsu Cornelius | | Med. Superintendent | Nkoranza Health Centre |  |
| 28. | Pru East | Ahmed Ramseyer | | DDHS | DHD |  |
| 29. |  | Ebenezer Gyamfi | | District Public Health Nurse | DHD |  |
| 30. |  | Kwasi Adjei | | Health Information Officer | DHD |  |
| 31. |  | Walter Kpo | | District Nutrition Officer | DHD |  |
| 32. |  | Patrick Kpodo | | Disease Control officer | DHD |  |
| **Oti Region** | | | | | | |
| 1. | Krachi West | George Agbeko | | Public Health Nurse | Krachi West Municipal Health Directorate |  |
| 2. |  | Raphael Akowuah | | Health Information Officer |  |  |
| 3. |  | Victor Ahiagba | | MDHS |  |  |
| 4. |  | Augustine Owusu-Addai | | Health Information Officer | Dr Bawuah Memorial Hospital |  |
| 5. |  | Patrick Bijabawan | | Planning Officer | Krachi West Municipal Assembly |  |
| 6. |  | Dr Theophilus Amoatey | | Medical Superintendent | Krachi West Municipal Hospital |  |
| 7. | Krachi Nchumuru | Peace Klatsu | | Public Health Nurse | Krachi Nchumuru District Health Directorate |  |
| 8. |  | Sampson Kpalam | | Health Information Officer |  |  |
| 9. |  | Stephen Totokari | | Health Promotion Officer |  |  |
| 10. |  | Hayford Zotorvie | | Physician Assistant |  |  |
| 11. |  | Abbass Yussif | | Programme Officer | CAAP-Ghana |  |
| 12. |  | Adabugra Akan Enye | | Planning Officer | Krachi Nchumuru District Assembly |  |
| 13. | Guan | Emmanuel Pewudie | | Programs Director | Mamacare-Ghana |  |
| 14. |  | Alhassan Issah | | Planning Officer | Guan District Assembly |  |
| 15. |  | Abdul Aziz Amamudu | | Ag. DDHS | Guan District Health Directorate |  |
| 16. |  | Rose Ayibor | | Public Health Nurse |  |  |
| 17. |  | Frederick Boateng | | Health Information Officer |  |  |
| 18. |  | Dise Okrah | | Nurse Practitioner | Likpe Polyclinic |  |
| 19. | Nkwanta North | Ivy Habla | | Health Information Officer | Nkwanta North District Health Directorate |  |
| 20. |  | Francis Botchway | | Public Health Nurse |  |  |
| 21. |  | Nicholas Tetteh | | Ag. DDHS |  |  |
| 22. |  | Clement Nteye | | Planning Officer | Nkwanta North District Assembly |  |
| 23. |  | Kenneth Bilijo | | Nutritionist | Somacas Medical Centre |  |
| 24. | Nkwanta South | Evans Attivor | | DDHS | Nkwanta South Health Directorate |  |
| 25. |  | Bright Akponorvi | | Public Health Nurse |  |  |
| 26. |  | Theresa Krampah | | Health Information Officer |  |  |
| 28. |  | Nelson Addy | | Senior Staff Nurse | Salifu CHPS Compound |  |
| 29. |  | Samuel Adu-Cocraine | | Programme Director | Tehayon Foundation |  |
| 30. |  | Hilarius Gadzey | | Medical Superintendent | Nkwanta South Municipal Hospital |  |

# Supplementary appendix 2: Attributes by dimension for each capacity assessed

## 3.1 Service provision capacity

**B 1: Better access to essential services**

**GUIDE BOX: This section captures status of access to essential services, from the aspects of physical, financial, socio-cultural factors. Please fill on the Likert scale the degree to which you agree/disagree for each intervention, for hospitals and primary level facilities. The ‘*80%’* mark used in measuring the attribute is mainly to serve as guide of ‘*almost all’* health facilities practicing or undertaking the specific attribute. The expectation is not that users will calculate the percentage of facilities delivering the entailed interventions, but by saying at least 80%, almost all facilities are contributing to the specific intervention If the information is not known or it does not apply in the SNU, enter ‘NA’. If the value is zero facilities contributing to the attribute, enter zero.**

| Vital Sign | **Attributes** | **Scoring**  *1 - Fully disagree*  *2 - Somewhat disagree*  *3 - Somewhat agree*  *4 - Fully agree* |
| --- | --- | --- |
|  |  |  |
| Physical access | Clinical staff (doctors, nurses, midwives) are available in at least 80% of facilities as per norms |  |
|  | Clinical support staff (pharmacists, etc) are available in at least 80% of facilities as per norms |  |
|  | Management and administrative staff are available in at least 80% of facilities as per norms |  |
|  | 80% of health facilities are available in the sub national unit as per country norms (eg. Within 5km of radius) |  |
|  | 80% of health facilities are physically accessible all year round (eg. No changes to access with changes in weather, transport, etc) |  |
|  | Outreach and mobile services are conducted in the last 3 months for at least 80% of hard to reach areas |  |
|  | Health commodities (tracer medicines) are available in 80% of facilities |  |
|  | Health commodities (vaccines ) are available in 80% of facilities |  |
|  | Health commodities (blood and blood products) are available in 80% of facilities |  |
|  | Health equipment (blood pressure apparatus, stethoscope, adult and infant scale, thermometer) are available in 80% of facilities |  |
| Financial access | Users of services are not expected to pay to access outpatient services |  |
|  | Users of services are not expected to pay to access inpatient services |  |
|  | Users of services are not expected to pay to access specialist clinics |  |
|  | There are mechanisms to identify and support indigent users to access services |  |
| Socio-cultural access | Social barriers to hindering women from accessing and using services are known and being addressed |  |
|  | Most of the young and adolescent girls are participating in formal schooling |  |
|  | Cultural practices that negatively impact women’s and children’s health are being addressed |  |
|  | Literary and education initiatives for the boy child are being implemented |  |
|  |  |  |
| **B2: Higher quality of care**  **GUIDE BOX: This section captures status of quality of health services, focusing on user experience, safety and effectiveness of care. Please fill on the Likert scale the degree to which you agree/disagree for each intervention, for the sub-national unit. The ‘*80%’* mark used in measuring the attribute is mainly to serve as guide of ‘*almost all’* health facilities practicing or undertaking the specific attribute. The expectation is not that users will calculate the percentage of facilities delivering the entailed interventions, but by saying at least 80%, almost all facilities are contributing to the specific intervention. If the information is not known or it does not apply in the SNU, enter ‘NA’. If the value is zero facilities contributing to the attribute, enter zero** | |  |
|  |  |  |
|  |  |  |
| \|  \| \| --- \|   . |  |  |
|  |  |  |
|  |  |  |
|  |  |  |
| User experiences | An independent process exists to review and follow up feedback on user experiences exists in at least 80% of facilities |  |
|  | Mechanisms to ensure users are treated with dignity as they receive care exist in at least 80% of facilities |  |
|  | Mechanisms to ensure confidentiality of users is assured as they receive care exist in at least 80% of facilities |  |
|  | Mechanisms to ensure users receive prompt attention when they seek care exist in at least 80% of facilities |  |
|  | Mechanisms to ensure users have choice of providers for their care exist |  |
|  | Mechanisms to ensure users have access to social support during care exist in at least 80% of facilities |  |
| User safety | Processes to prevent, monitor and act on medical errors is in place in at least 80% of facilities |  |
|  | Mechanisms to identify, and respond to adverse events following care provision is functional in at least 80% of facilities |  |
|  | Health facilities have in place vigilance and awareness initiatives involving users, to eliminate harm to users |  |
|  | Surveys of patient safety are conducted at least once every 2 years in up to 80% of facilities |  |
|  | At least 80% of health facilities are monitoring the 8 common causes of poor safety: (1) medical errors, (2) health care associated infections, (3) unsafe surgical procedures, (4) unsafe injections, (5) diagnostic errors, (6)unsafe transfusions, (7) sepsis and (8) blood clots |  |
| Effectiveness of care | At least 80% of hospitals have a functional therapeutics committee |  |
|  | At least 80% of hospitals have results of clinical audits they have conducted in the past 1 year |  |
|  | Standard management guidelines are used across at least 80% of facilities |  |
|  | At least 80% of clinical staff have received continuing professional development focusing on their area of work in the past 1 year |  |
|  | At least 80% of hospitals have clinical governance committees / forums involving patient groups to ensure excellent clinical care |  |
|  |  |  |
| **B3: Effective demand for essential services** | |  |
|  |  |  |
| \|  \| \| --- \| | **GUIDE BOX: This section captures demand of health services, focusing on individual’s healthy action and health seeking behaviours, for all age cohorts. Please fill on the Likert scale the degree to which you agree/disagree for each intervention, for the sub-national unit. The ‘*80%’* mark used in measuring the attribute is mainly to serve as guide of ‘*almost all’* health facilities practicing or undertaking the specific attribute. The expectation is not that users will calculate the percentage of facilities delivering the entailed interventions, but by saying at least 80%, almost all facilities are contributing to the specific intervention If the information is not known or it does not apply in the SNU, enter ‘NA’. If the value is zero facilities contributing to the attribute, enter zero** |  |
|  |  |  |
|  |  |  |
|  |  |  |
|  |  |  |
| Individual healthy action | Programs for promoting health and wellbeing for individuals are available for at least 80% of pregnant mothers and newborns |  |
|  | Programs for promoting health and wellbeing for individuals are available for at least 80% of under 5 year old children |  |
|  | Programs for promoting health and wellbeing for individuals are available for at least 80% of adolescent females |  |
|  | Programs for promoting health and wellbeing for individuals are available for at least 80% of adolescent males |  |
|  | Programs for promoting health and wellbeing for individuals are available for at least 80% of adults |  |
|  | Programs for promoting health and wellbeing for individuals are available for at least 80% of elderly persons |  |
| Health seeking behaviors | Processes to monitor and encourage appropriate health seeking behavior are available for at least 80% of pregnant mothers and newborns |  |
|  | Processes to monitor and encourage appropriate health seeking behavior are available for at least 80% of under 5 year old children |  |
|  | Processes to monitor and encourage appropriate health seeking behavior are available for at least 80% of adolescent females |  |
|  | Processes to monitor and encourage appropriate health seeking behavior are available for at least 80% of adolescent males |  |
|  | Processes to monitor and encourage appropriate health seeking behavior are available for at least 80% of adults |  |
|  | Processes to monitor and encourage appropriate health seeking behavior are available for at least 80% of elderly persons |  |
|  |  |  |
|  |  |  |
| **B4: Robust resilience to shock events** | |  |
| **GUIDE BOX: This section captures resilience status of health systems to shock events, focusing on targeted and inherent resilience. Please fill on the Likert scale the degree to which you agree/disagree for each intervention, for the sub-national unit. If the information is not known or it does not apply in the SNU, enter ‘NA’. If the value is zero facilities contributing to the attribute, enter zero** | . |  |
| \|  \| \| --- \| |  |  |
|  |  |  |
| Targeted resilience | There exists appropriate capacity for Leadership and governance for targeted shock events for: |  |
|  | *·      Legislative, policy and dedicated financing* |  |
|  | *·      IHR coordination, communication and advocacy* |  |
|  | *·      Monitoring and reporting Antimicrobial resistance (AMR)* |  |
|  | *·      Enforcement of Food safety regulations* |  |
|  | There exists appropriate capacity for monitoring level of preparedness for*:* |  |
|  | *·      Detection and control of Zoonotic disease* |  |
|  | *·      Effecting Biosafety and biosecurity regulations* |  |
|  | *·      Monitoring and enforcing Immunization according to IHR including cross border regulations (PoE)* |  |
|  | There exists appropriate capacity for laboratory systems to detect health risk in areas of: |  |
|  | *·      Safety and standards* |  |
|  | *·      Linking public health with law and multisectoral rapid response* |  |
|  | *·      Medical countermeasures and personnel deployment* |  |
|  | *·      Real-time surveillance of infectious diseases or health hazards* |  |
|  | There exists adequate capacity for coordination and communication in response to potential threats, focusing on: |  |
|  | *·      Requisite skilled workforce to monitor and detect health risk* |  |
|  | *·      Established Emergency operations coordination centre* |  |
|  | *·      Risk communication* |  |
|  | *·      Monitor, Manage and Report Radiation emergencies* |  |
|  |  |  |
| Inherent resilience | Awareness of the system |  |
|  | *·      There is documented up to date (under 1 year old) mapping of the health system assets – specifically staff, infrastructure, commodities* |  |
|  | *·      There is documented up to date (under 1 year old) mapping of potential shocks – covering acute and chronic disease, environmental, economic, and political shocks* |  |
|  | *·      There is a functional surveillance network reporting both weekly on notifiable diseases, and monthly on health system capacity changes* |  |
|  | *·      Simulation exercises have been conducted in the past 1 year, assessing capacity to respond to potential shock events of highest risk of occurrence* |  |
|  | *·      There are agreed standard operating procedures for ensuring functional staff, supplies and infrastructure in the event of a shock event* |  |
|  | Diversity in provision of services |  |
|  | *·      Health facilities have functional therapeutics committees that are monitoring rare / uncommon events impacting service provision* |  |
|  | *·      There are no stock outs in the past 1 year for common supportive drugs and supplies used in emergency (Oxygen, analgesics, PPEs, and other supportive supplies)* |  |
|  | *·      Health facilities have micro-plans, to take essential services to hard to reach populations in their areas of responsibility* |  |
|  | *·      Health facilities are aware of the range of essential services they are expected to provide, and have plans to expand their capacity to provide these* |  |
|  | *·      Health facilities are utilizing multiple service delivery approaches: fixed sites, outreaches, mobile clinics, e-referrals, etc to take services to their population* |  |
|  | Versatility and self-regulation capacity |  |
|  | *·      The primary care (front line) facilities have the needed epidemiology and other technical skills to identify and isolate health threats* |  |
|  | *·      There are standard operating procedures to allow health facility staff to repurpose their infrastructure, staff and medical supplies when facing potential threats* |  |
|  | *·      There exist processes to guide facilities on how to identify and shield staff, infrastructure and medical supplies for continuing essential services provision during threats* |  |
|  | *·      Mechanisms exist for coordinating additional capacities (staff, infrastructure, medical supplies) mobilized to respond to threats* |  |
|  | *·      Health facility staff have the required decision, authority and protocols to initiate action and spend funds in event of shock events* |  |
|  | Mobilization and deployment capacity |  |
|  | ·      *There are functional mechanisms for communication and engagement with non-public health providers working in the area of responsibility of public facilities – such as private sector, NGOs, CSOs, and others* |  |
|  | ·      *There are functional mechanisms for communication and engagement with community groups in the area of responsibility of public facilities* |  |
|  | ·      *There are functional mechanisms for communication and engagement with other health related sectors in the area of responsibility of public facilities – such as agriculture, water, security, etc* |  |
|  | ·      *There are pre-agreed mechanisms for sharing of personnel, funds and capacities amongst stakeholders working within their areas of responsibility of facilities* |  |
|  | ·      *Public, and private sources of additional capacities (staff, infrastructure, medical supplies) for surge capacity are known and procedures to bring these on board are available* |  |
|  | Transformation capacity |  |
|  | ·      *There is regularly updated information on the level of functionality of the health system* |  |
|  | ·      *There are agreed protocols to guide absorption of resources and skills mobilized during a response to an event into the routine system* |  |
|  | ·      *There are protocols to constantly monitor essential service provision during a shock event exist* |  |
|  | ·      *There is guidance on comprehensive recovery planning based on assessment, and investment across the health system* |  |
|  | ·      *Process documentation and intelligence generation is planned for shock events, and a repository of such lessons exists* |  |
|  |  |  |
| **C)    ASSESSING THE MANAGEMENT CAPACITY** | |  |
|  | .  **GUIDE BOX: This section assesses the management capacity of SNUs, focused on the 7S framework. Please fill on the Likert scale the degree to which you agree/disagree for each intervention, in the sub-national unit. If the information is not known or it does not apply in the SNU, enter ‘NA’. If the value is zero facilities contributing to the attribute, enter zero** |  |
|  | . |  |
|  | . |  |
|  |  |  |
|  |  |  |
| Structure | All hospitals offer the four categories of hospital services |  |
|  | *A centre of excellence for specialized and specific complementary services* |  |
|  | *An in-service training centre for staff in related primary care facilities* |  |
|  | *Pre-service and internship training for defined staff cadres* |  |
|  | *A research centre for generating and using evidence in the area of responsibility* |  |
|  | At least 80% of the primary care facilities have the expected 10 service areas functional: |  |
|  | *(1) Outpatient general* |  |
|  | *(2) outpatient emergency (Accident and Emergency)* |  |
|  | *(3) outpatient operative services* |  |
|  | *(4) outpatient specialized clinics (e.g. ANC)* |  |
|  | *(5) investigative/laboratory* |  |
|  | *(6) inpatient and admission services – male and female* |  |
|  | *(7) disease prevention services* |  |
|  | *(8) health promotion services* |  |
|  | *(9) rehabilitative services* |  |
|  | *(10) palliative care* |  |
|  | There are not more than 2 levels of care between beneficiaries and the highest quality services (Beneficiary -> ***one level*** -> specialist care) |  |
| Strategy | Each health facility has defined areas of responsibility with a fixed population who’s health it is responsible for |  |
|  | The sub national unit has clearly defined coverage targets for UHC, and health security to guide its actions |  |
|  | The sub national unit has clearly elaborated essential health interventions across public health functions by level of care, and age cohort |  |
|  | The sub national unit has explicit targets for improving capacities relating to better access, quality, demand and resilience of the system |  |
|  | The sub national unit has a medium-term strategy for the progressive realization of targets for UHC & health security |  |
|  | The sub national unit has an annual operational planning process that involves all its programs and partners |  |
| Systems | The sub national unit conducts regular supportive supervision of health facilities assessing licensure and accreditation |  |
|  | At least 80% of primary care facilities have models of service delivery to take services into the community (home visits, community outreaches, community events, etc) |  |
|  | At least 80% of clients in hospitals are referred from primary care facilities |  |
|  | All hospitals are utilizing online consultations for their beneficiaries to improve capacity and access |  |
|  | All hospitals have systems for effective referral for: |  |
|  | *patient movement (ambulance services),* |  |
|  | *sample movement (laboratory sample extraction),* |  |
|  | *information movement (e-consultations)* |  |
|  | *specialist movement (non-resident specialist clinics)* |  |
| Style | Health facilities with low (under 50%) expected HR and infrastructure norms have managers with **strategic leadership attributes**: Individually competent and visionary with long term strategic direction |  |
|  | Health facilities with average (about 50%) HR and infrastructure norms have managers with **transactional leadership attributes**: Able to handle multiple complex activities concurrently and craft appropriate organizational cultures |  |
|  | Health facilities with above average (up to 80%) of HR and infrastructure norms have managers with **democratic leadership attributes**: Competent in managing people, and able to coach and nurture other staff to take on responsibilities |  |
|  | Health facilities with expected or more (over 80%) of HR and infrastructure norms have managers with **transformational leadership attributes**: Able to effectively manage multiple teams |  |
| Skills | At least 80% of primary care facilities have staff with technical skills to treat, rehabilitate and provide palliative care for users with: |  |
|  | *HIV/AIDS* |  |
|  | *Tuberculosis* |  |
|  | *Malaria* |  |
|  | *Hypertension* |  |
|  | *Diabetes* |  |
|  | *Cardiovascular disease* |  |
|  | *Cancer* |  |
|  | *Mental Health* |  |
|  | At least 80% of primary care facilities have staff with the following competencies |  |
|  | *Communication* |  |
|  | *Professionalism* |  |
|  | *Business skills* |  |
|  | *Knowledge of health systems development* |  |
|  | *Coordination of inputs (HR, infrastructure, medicines)* |  |
|  | All hospitals have staff with technical skills to treat, rehabilitate and provide palliative care for users with: |  |
|  | *HIV/AIDS* |  |
|  | *Tuberculosis* |  |
|  | *Malaria* |  |
|  | *Hypertension* |  |
|  | *Diabetes* |  |
|  | *Cardiovascular disease* |  |
|  | *Cancer* |  |
|  | *Mental Health* |  |
|  | All hospitals have staff with the following competencies |  |
|  | *Communication* |  |
|  | *Professionalism* |  |
|  | *Business skills* |  |
|  | *Knowledge of health systems development* |  |
|  | *Coordination of inputs (HR, infrastructure, medicines)* |  |
| Staff | The management function has the required complement of staff to enable its functions. Teams needed include: |  |
|  | *Management team for leading delivery of services (includes programs)* |  |
|  | *Therapeutics committee for monitoring care standards and outcomes* |  |
|  | *Coordination committee for coordination of stakeholders* |  |
|  | *Oversight team for accountability, voice and engagement of users* |  |
| Shared values | At least 80% of primary care facilities have management teams with appropriate core values of |  |
|  | *Passion and drive: Ability to perform roles with purpose, pride, and a positive attitude* |  |
|  | *Honesty and integrity: Ability to uphold ethics and principles with every action and every decision* |  |
|  | *Efficiency: Ability to achieve quality results with the least possible resources* |  |
|  | *Reliability and dependability: Ability to do what is expected, all the time* |  |
|  | *Commitment: dedicated to the long term success of the sub national unit, specifically the health of its population* |  |
|  | *Innovative: Staff are ready, and embrace change for the better* |  |
|  | All hospitals have management teams with appropriate core values of |  |
|  | *Passion and drive: Ability to perform roles with purpose, pride, and a positive attitude* |  |
|  | *Honesty and integrity: Ability to uphold ethics and principles with every action and every decision* |  |
|  | *Efficiency: Ability to achieve quality results with the least possible resources* |  |
|  | *Reliability and dependability: Ability to do what is expected, all the time* |  |
|  | *Commitment: dedicated to the long term success of the sub national unit, specifically the health of its population* |  |
|  | *Innovative: Staff are ready, and embrace change for the better* |  |
|  |  |  |
| **D)    ASSESSING THE OVERSIGHT CAPACITY** | |  |
|  | . |  |
|  | . |  |
|  | . |  |
| . |  |  |
|  |  |  |
| \ |  |  |
| **Authority**  *The leadership has the needed impicit and explicit ability to make decisions relating to health resources in the area of responsibility of the unit* | There is appropriate decentralization legal framework that allows effective decision space for local management of funds and investments |  |
|  | The decision making authority of the health leadership is recognized and respected by other stakeholders, including |  |
|  | *Sub national political and administrative structures* |  |
|  | *National level health institutions such as MOH* |  |
|  | *Sub national stakeholders ssuch as civil society and NGOs* |  |
|  | *Sub national private sector providers* |  |
|  | The health leaders have the required skills to make use of the availabel decision space and exercise its explicit mandate |  |
| **Organizational structure**  *The existing structure is an appropriate translation of the existing authority, to facilitate implementation of oversight functions and expectations* | An explicit organogram for the sub national unit exists, and is recent (under 3 years old) |  |
|  | The health leadership is responsible for the development and operationalization of the organogram |  |
|  | The expected health functions of the sub national unit are accurately mapped with the approved organogram – with minimal redundunt positions |  |
|  | Each member of the leadership has clear terms of reference, and their responsibilities are explicitly outlined to the organogram |  |
|  | There are functional mechanisms (e.g. committees) to coordinate the functions of the organogram, maximizing synergies, minimizing overlaps and eliminating gaps in responsibility |  |
| **Policy and strategic guidance**  *A clear long and medium term direction exists, aligned with the overall government and health sector directions* | The sub national unit has a clear policy direction guiding its long term (over 5 years) health development  **GUIDE BOX: This section captures oversight capacity, assessing attributes for authority, organisational structure, policy and strategic guidance, technical and social accountability, legal and regulatory frameworks, stakeholders’ engagement, integrity and public confidence. Please fill on the Likert scale the degree to which you agree/disagree for each intervention, in the sub-national unit. If the information is not known or it does not apply in the SNU, enter ‘NA’. If the value is zero facilities contributing to the attribute, enter zero** |  |
|  | The sub national unit has a clear strategic direction guiding its medium term (3 – 5 years) health development |  |
|  | The sub national unit has an annual operational plan consolidated from operational plans for all its service provision units |  |
|  | The strategic direction of the sub national unit is aligned (in time and content) with that of the political and aadministrative unit it works within |  |
|  | The policy and strategic direction of the sub national unit are aligned (in time and content) with that of the overall health sector |  |
| **Technical accountability**  *Mechanisms to ensure answerability of health leadership to the health agenda* | Regular quarterly reviews of operational implementation are consistently carried out |  |
|  | Once a year performance monitoring review is conducted, reviewing progress against planned activities of the sub national unit |  |
|  | The sub national unit has a process to analyse health data in order to have realtime health intelligence on the state of service provision and capacity |  |
|  | Strategic review of the sector progress towards its goals has been conducted within the passt 5 years |  |
| **Social accountability:**  *Mechanisms to ensure answerability of health leadership to the public* | Once a year health summit is convened involving the public, partners, civil society, private actors and other stakeholders to share progress by all stakeholders |  |
|  | The public are involved in the decision making process for public ressources (open budgeting) |  |
|  | Reports on progress with health are shared with the public for scrutiny and input |  |
|  | Information on all resources – public, donor and private – are available and shared with all health actors, particularly the public |  |
| **Legal and regulatory mechanisms:**  *Required formal and informal instruments that give mandate to act* | Health acts exist, covering service delivery, plus management of the health workforce, infrastructure deployment and medical products use |  |
|  | Required local regulations exist to implement the health Acts |  |
|  | There is appropriate enforcement capacity for adherence to the regulatory and legal instruments |  |
|  | The health leadership is aware of, and has oriented all the health management on the informal societal norms that affect health service provision – e.g. on values of the placenta, gender roles in health, etc |  |
| **Stakeholder engagement:**  *Mandate, responsibilities of stakeholders in health are clear and monitored* | Roles of public, private and external partners in the health agenda are clearly elaborated |  |
|  | A partnership instrument that consolidated responsibilities, defines how they are enforced and actions when they are not is in place (a compact / Memorandum of Understanding / Code of Conduct / etc) |  |
|  | A functional public-private partnership exists that ensures complementary actions are in palce to deliver on the health agenda is in place |  |
|  | Regular meetings are held with external and non-public stakeholders to review progress with harmonization and joint working arrangements |  |
| **Integrity & public confidence:**  *Active processes are in place to build the public trust and respect in the sector* | Each service provision unit has a functional means to get views on the sector from the public (hotlines, anticorruption boxes, etc) |  |
|  | The sub national unit has a team dedicated to engaging with the public on a constant basis, to ensure it is constantly aware of public views |  |
|  | The sub national unit has a fully functional process to constantly capture views of the public on its integrity |  |
|  | The sub national unit has a culture of taking decisive and corrective action when its reputation and integrity is compromised – with no exceptions |  |

# Supplementary appendix 3: Mean scores by dimension for each district assessed

## Oversight capacity dimensions and scores

| District | Authority | Stewardship | Policy and strategy | Technical accountability | Social accountability | Legal & regulatory framework | Stakeholder engagement | Integrity & public confidence |
| --- | --- | --- | --- | --- | --- | --- | --- | --- |
| JUAN | 75.00 | 100.00 | 100.00 | 100.00 | 100.00 | 100.00 | 93.75 | 100.00 |
| NKWANTA SOUTH | 100.00 | 100.00 | 85.00 | 87.50 | 87.50 | 100.00 | 93.75 | 93.75 |
| NKWANTA NORTH | 91.67 | 85.00 | 75.00 | 93.75 | 56.25 | 75.00 | 75.00 | 50.00 |
| TANO NORTH | 79.17 | 95.00 | 100.00 | 93.75 | 93.75 | 100.00 | 93.75 | 93.75 |
| ASUTIFI SOUTH | 83.33 | 100.00 | 100.00 | 87.50 | 68.75 | 100.00 | 93.75 | 81.25 |
| NKORANZA SOUTH | 95.83 | 70.00 | 90.00 | 93.75 | 75.00 | 100.00 | 93.75 | 81.25 |
| BUNKPURUGU NAKPANDURI | 83.33 | 75.00 | 65.00 | 75.00 | 43.75 | 50.00 | 56.25 | 62.50 |
| KRACHI WEST | 95.83 | 100.00 | 100.00 | 100.00 | 62.50 | 100.00 | 100.00 | 87.50 |
| KRACHI NCHUMURU | 100.00 | 100.00 | 100.00 | 100.00 | 100.00 | 100.00 | 87.50 | 75.00 |
| ASUNAFO SOUTH | 95.83 | 100.00 | 100.00 | 100.00 | 100.00 | 100.00 | 81.25 | 81.25 |
| TANO SOUTH | 87.50 | 80.00 | 100.00 | 93.75 | 68.75 | 93.75 | 75.00 | 56.25 |
| ASUTIFI NORTH | 95.83 | 95.00 | 95.00 | 87.50 | 87.50 | 87.50 | 93.75 | 87.50 |
| KINTAMPO NORTH MUNICIPAL | 100.00 | 100.00 | 100.00 | 100.00 | 87.50 | 93.75 | 75.00 | 93.75 |
| TECHIMAN MUNICIPAL | 79.17 | 95.00 | 70.00 | 100.00 | 68.75 | 62.50 | 62.50 | 56.25 |
| PRU | 95.83 | 100.00 | 55.00 | 100.00 | 75.00 | 100.00 | 100.00 | 87.50 |
| YUNYOO-NASUAN | 95.83 | 95.00 | 90.00 | 100.00 | 56.25 | 87.50 | 93.75 | 75.00 |
| CHEREPONI | 91.67 | 100.00 | 100.00 | 100.00 | 100.00 | 87.50 | 100.00 | 100.00 |
| EAST MAMPRUSI | 75.00 | 100.00 | 85.00 | 87.50 | 87.50 | 62.50 | 68.75 | 68.75 |
| GA EAST | 83.33 | 85.00 | 85.00 | 87.50 | 81.25 | 81.25 | 43.75 | 75.00 |
| NINGO PRAMPRAM | 87.50 | 90.00 | 100.00 | 100.00 | 43.75 | 81.25 | 68.75 | 62.50 |
| BIA EAST | 95.83 | 100.00 | 100.00 | 93.75 | 43.75 | 100.00 | 81.25 | 87.50 |
| BODI | 91.67 | 95.00 | 95.00 | 100.00 | 100.00 | 100.00 | 100.00 | 81.25 |
| MAMPRUGU MOAGDURI | 95.83 | 90.00 | 85.00 | 81.25 | 75.00 | 100.00 | 93.75 | 68.75 |
| AOWIN | 100.00 | 95.00 | 95.00 | 100.00 | 68.75 | 81.25 | 87.50 | 62.50 |
| WEST MAMPRUSI MUNICIPAL | 66.67 | 100.00 | 100.00 | 100.00 | 100.00 | 100.00 | 100.00 | 100.00 |
| BOLE | 100.00 | 100.00 | 80.00 | 87.50 | 50.00 | 81.25 | 75.00 | 75.00 |
| NORTH EAST GONJA | 91.67 | 100.00 | 100.00 | 87.50 | 93.75 | 93.75 | 100.00 | 75.00 |
| EAST GONJA | 100.00 | 100.00 | 100.00 | 100.00 | 81.25 | 100.00 | 100.00 | 100.00 |
| CENTRAL GONJA | 87.50 | 100.00 | 100.00 | 100.00 | 100.00 | 100.00 | 100.00 | 100.00 |
| NORTH GONJA | 100.00 | 100.00 | 100.00 | 100.00 | 100.00 | 100.00 | 100.00 | 100.00 |
| SAWLA-TUNA-KALBA | 100.00 | 95.00 | 100.00 | 100.00 | 93.75 | 93.75 | 87.50 | 100.00 |
| WEST GONJA | 100.00 | 100.00 | 100.00 | 100.00 | 87.50 | 93.75 | 100.00 | 100.00 |
| SEFWI AKONTOMBRA | 95.83 | 65.00 | 80.00 | 81.25 | 81.25 | 75.00 | 87.50 | 100.00 |

## Management capacity dimensions

| District | Structure | Strategy | Systems | Style | Skills | Staff | Values |
| --- | --- | --- | --- | --- | --- | --- | --- |
| JUAN | 50.00 | 100.00 | 37.50 | 87.50 | 44.23 | 81.25 | 50.00 |
| NKWANTA SOUTH | 95.00 | 95.83 | 84.38 | 87.50 | 90.38 | 100.00 | 100.00 |
| NKWANTA NORTH | 43.33 | 79.17 | 46.88 | 56.25 | 28.85 | 56.25 | 41.67 |
| TANO NORTH | 96.67 | 79.17 | 90.63 | 93.75 | 91.35 | 100.00 | 100.00 |
| ASUTIFI SOUTH | 88.33 | 87.50 | 84.38 | 75.00 | 84.62 | 93.75 | 100.00 |
| NKORANZA SOUTH | 58.33 | 87.50 | 65.63 | 75.00 | 76.92 | 81.25 | 85.42 |
| BUNKPURUGU NAKPANDURI | 66.67 | 75.00 | 53.13 | 75.00 | 58.65 | 56.25 | 75.00 |
| KRACHI WEST | 78.33 | 87.50 | 90.63 | 75.00 | 83.65 | 81.25 | 75.00 |
| KRACHI NCHUMURU | 46.67 | 100.00 | 68.75 | 81.25 | 38.46 | 100.00 | 47.92 |
| ASUNAFO SOUTH | 91.67 | 87.50 | 84.38 | 93.75 | 89.42 | 75.00 | 95.83 |
| TANO SOUTH | 63.33 | 87.50 | 78.13 | 81.25 | 85.58 | 81.25 | 81.25 |
| ASUTIFI NORTH | 61.67 | 91.67 | 78.13 | 87.50 | 82.69 | 87.50 | 83.33 |
| KINTAMPO NORTH MUNICIPAL | 75.00 | 100.00 | 75.00 | 100.00 | 94.23 | 100.00 | 100.00 |
| TECHIMAN MUNICIPAL | 63.33 | 100.00 | 59.38 | 68.75 | 68.27 | 62.50 | 79.17 |
| PRU | 78.33 | 100.00 | 75.00 | 87.50 | 92.31 | 56.25 | 81.25 |
| YUNYOO-NASUAN | 60.00 | 100.00 | 59.38 | 50.00 | 38.46 | 62.50 | 50.00 |
| CHEREPONI | 88.33 | 100.00 | 87.50 | 93.75 | 86.54 | 75.00 | 100.00 |
| EAST MAMPRUSI | 73.33 | 87.50 | 53.13 | 62.50 | 80.77 | 50.00 | 95.83 |
| GA EAST | 68.33 | 50.00 | 46.88 | 87.50 | 71.15 | 81.25 | 70.83 |
| NINGO PRAMPRAM | 81.67 | 91.67 | 59.38 | 75.00 | 75.00 | 56.25 | 79.17 |
| BIA EAST | 83.33 | 95.83 | 46.88 | 100.00 | 44.23 | 100.00 | 50.00 |
| BODI | 51.67 | 83.33 | 25.00 | 75.00 | 38.46 | 68.75 | 47.92 |
| MAMPRUGU MOAGDURI | 48.33 | 100.00 | 46.88 | 75.00 | 83.65 | 25.00 | 100.00 |
| AOWIN | 51.67 | 91.67 | 78.13 | 100.00 | 79.81 | 87.50 | 89.58 |
| WEST MAMPRUSI MUNICIPAL | 90.00 | 100.00 | 90.63 | 100.00 | 98.08 | 100.00 | 100.00 |
| BOLE | 78.33 | 87.50 | 68.75 | 75.00 | 75.96 | 75.00 | 75.00 |
| NORTH EAST GONJA | 65.00 | 87.50 | 78.13 | 75.00 | 97.12 | 87.50 | 97.92 |
| EAST GONJA | 95.00 | 100.00 | 81.25 | 93.75 | 100.00 | 100.00 | 100.00 |
| CENTRAL GONJA | 100.00 | 100.00 | 81.25 | 75.00 | 82.69 | 75.00 | 100.00 |
| NORTH GONJA | 55.00 | 79.17 | 25.00 | 68.75 | 41.35 | 87.50 | 50.00 |
| SAWLA-TUNA-KALBA | 96.67 | 100.00 | 93.75 | 87.50 | 100.00 | 93.75 | 100.00 |
| WEST GONJA | 86.67 | 87.50 | 90.63 | 62.50 | 81.73 | 81.25 | 75.00 |
| SEFWI AKONTOMBRA | 65.00 | 95.83 | 62.50 | 100.00 | 78.85 | 81.25 | 97.92 |

## Service provision capacity dimensions

| District | Access to essential services | Quality of care | Demand for essential services | Resilience to shock events |
| --- | --- | --- | --- | --- |
| JUAN | 41.07 | 71.88 | 100.00 | 81.88 |
| NKWANTA SOUTH | 79.46 | 90.63 | 95.83 | 80.63 |
| NKWANTA NORTH | 41.96 | 50.00 | 66.67 | 58.13 |
| TANO NORTH | 79.46 | 98.44 | 89.58 | 87.50 |
| ASUTIFI SOUTH | 77.68 | 92.19 | 100.00 | 83.13 |
| NKORANZA SOUTH | 69.64 | 54.69 | 100.00 | 65.63 |
| BUNKPURUGU NAKPANDURI | 69.64 | 56.25 | 56.25 | 51.25 |
| KRACHI WEST | 93.75 | 87.50 | 100.00 | 77.50 |
| KRACHI NCHUMURU | 48.21 | 82.81 | 100.00 | 92.50 |
| ASUNAFO SOUTH | 83.93 | 92.19 | 83.33 | 91.25 |
| TANO SOUTH | 75.89 | 62.50 | 100.00 | 73.75 |
| ASUTIFI NORTH | 83.93 | 78.13 | 81.25 | 78.75 |
| KINTAMPO NORTH MUNICIPAL | 83.04 | 78.13 | 97.92 | 85.00 |
| TECHIMAN MUNICIPAL | 82.14 | 78.13 | 91.67 | 61.25 |
| PRU | 75.00 | 76.56 | 95.83 | 80.00 |
| YUNYOO-NASUAN | 41.96 | 67.19 | 81.25 | 68.13 |
| CHEREPONI | 81.25 | 85.94 | 100.00 | 88.13 |
| EAST MAMPRUSI | 73.21 | 79.69 | 75.00 | 75.00 |
| GA EAST | 74.11 | 68.75 | 85.42 | 51.88 |
| NINGO PRAMPRAM | 68.75 | 62.50 | 87.50 | 75.00 |
| BIA EAST | 58.93 | 76.56 | 100.00 | 88.13 |
| BODI | 42.86 | 70.31 | 83.33 | 70.63 |
| MAMPRUGU MOAGDURI | 40.18 | 60.94 | 85.42 | 61.25 |
| AOWIN | 59.82 | 85.94 | 87.50 | 72.50 |
| WEST MAMPRUSI MUNICIPAL | 88.39 | 96.88 | 100.00 | 98.13 |
| BOLE | 70.54 | 71.88 | 91.67 | 65.00 |
| NORTH EAST GONJA | 34.82 | 57.81 | 87.50 | 77.50 |
| EAST GONJA | 87.50 | 98.44 | 100.00 | 98.75 |
| CENTRAL GONJA | 84.82 | 96.88 | 100.00 | 94.38 |
| NORTH GONJA | 36.61 | 60.94 | 100.00 | 79.38 |
| SAWLA-TUNA-KALBA | 80.36 | 81.25 | 89.58 | 91.25 |
| WEST GONJA | 72.32 | 85.94 | 89.58 | 83.75 |
| SEFWI AKONTOMBRA | 91.96 | 78.13 | 60.42 | 69.38 |
